# Supplementary material for: Towards early diagnosis and screening of Alzheimer’s disease using frequency locked whispering gallery mode microtoroids
Source: NPJ Biosens. 2024 Aug 28;1(1):9. doi: 10.1038/s44328-024-00009-8 (PMC12173943; doi:10.1038/s44328-024-00009-8)
Supplement: Supplementary file 1 — Supplementary information [file 44328_2024_9_MOESM1_ESM.pdf]

Supplementary Information for:

Towards Early Diagnosis and Screening of Alzheimer's Disease Using Frequency Locked Whispering Gallery Mode Microtoroids

Adley Gin[1], Phuong-Diem Nguyen[2], Geidy Serrano[3,7], Gene E. Alexander,[4,5,6,7,8] and Judith Su[1,2*]

[1]*Wyant College of Optical Sciences, The University of Arizona, Tucson, AZ, USA*

[2]*Department of Biomedical Engineering, The University of Arizona, Tucson, AZ, USA*

[3]*Barrow Neurological Institute, Phoenix, AZ, USA*

[4]*Department of Psychology, The University of Arizona, Tucson, AZ, USA*

[5]*Department of Psychiatry, The University of Arizona, Tucson, AZ, USA*

[6]*Neuroscience and Physiological Sciences Graduate Interdisciplinary Programs, The University of Arizona, Tucson, AZ, USA*

[7]*Evelyn F. McKnight Brain Institute, The University of Arizona, Tucson, AZ, USA*

[8]*Arizona Alzheimer's Consortium, Phoenix, AZ, USA*

**Address correspondence to: judy@optics.arizona.edu*

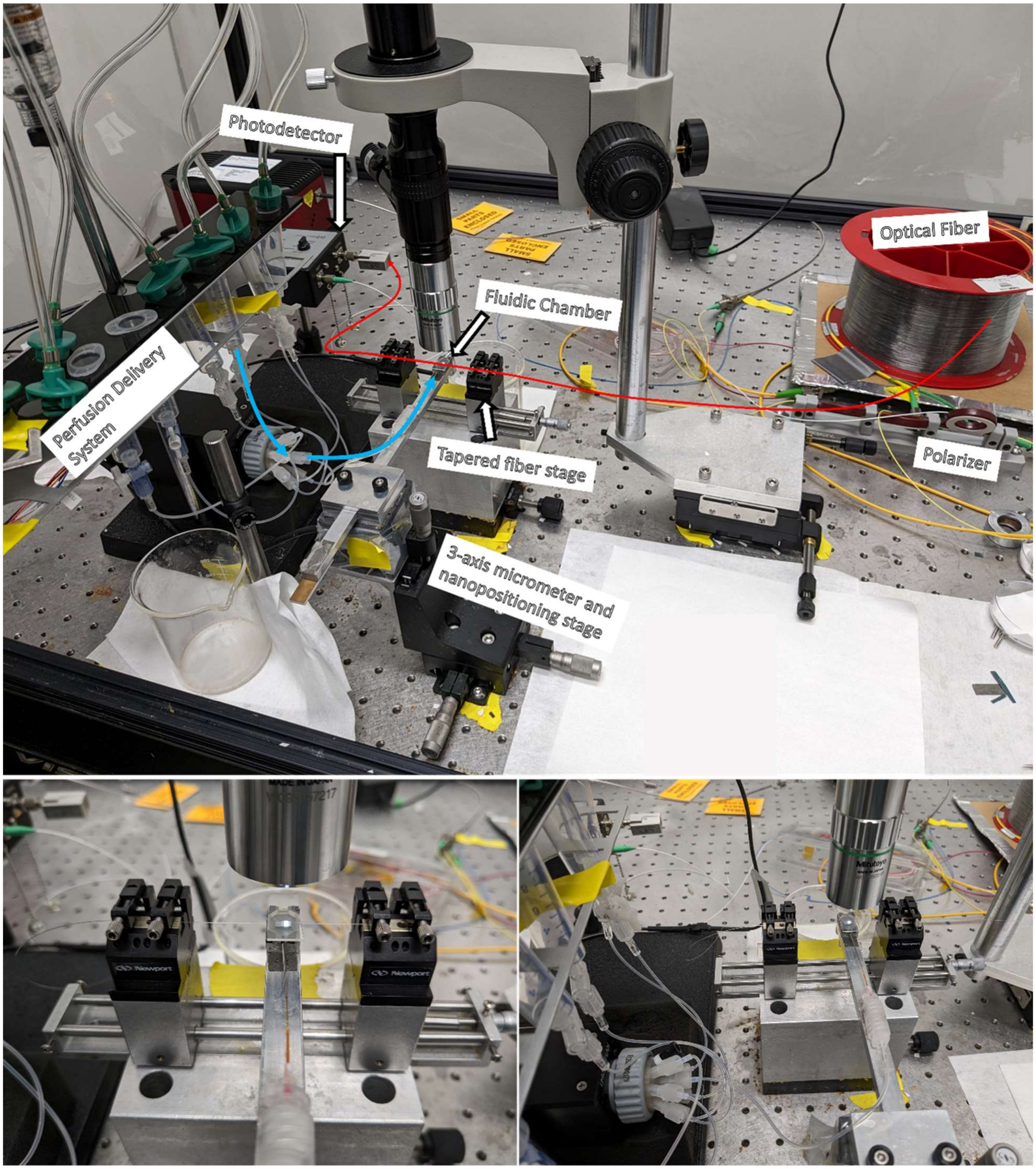

Supplementary Figure 1. FLOWER experimental setup. The red line depicts the direction of the laser traveling through the optical fiber, into tapered portion, and then into the photodetector. The blue line depicts the flow of liquid sample from the pressurized perfusion system, through the 8-channel rotary valve, then through a 100 μm perfusion pencil tip into the fluidic chamber. The toroid chip is affixed into the fluidic chamber using double sided tape. The fluidic chamber is stuck onto the end of a metal rod, which is mounted onto the 3-axis micrometer and nanopositioning stage. A cover glass is placed on top of the fluidic chamber to contain the fluid.

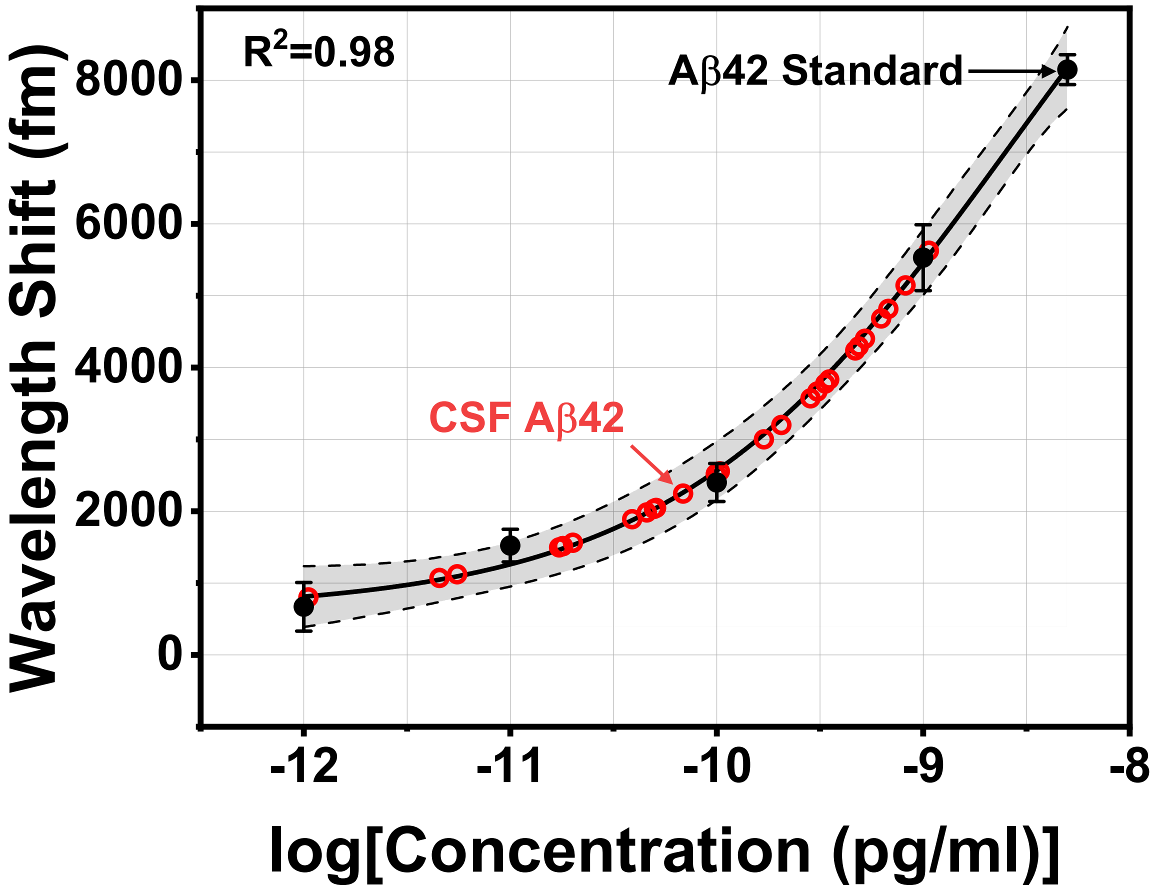

Supplementary Figure 2. Aβ42 Standard calibration curve with mean and standard deviation. Error bars for each point were obtained from 3 separate experiments, except for the highest concentration (5000 pg/ml) which has only 2 repeats. Fitting parameters with standard error: $A_1=611 \pm 325$, $A_2=13618 \pm 4645$, $Logx_0=-8.57 \pm 0.580$, $p=0.526 \pm 0.145$. The dissociation constant, K_D, of the 12F4-Aβ42-6E10 sandwich assay was calculated to be ~3 ng/mL, which is around the same order of magnitude reported by surface plasmon resonance and analytical ultracentrifugation assays.[1]

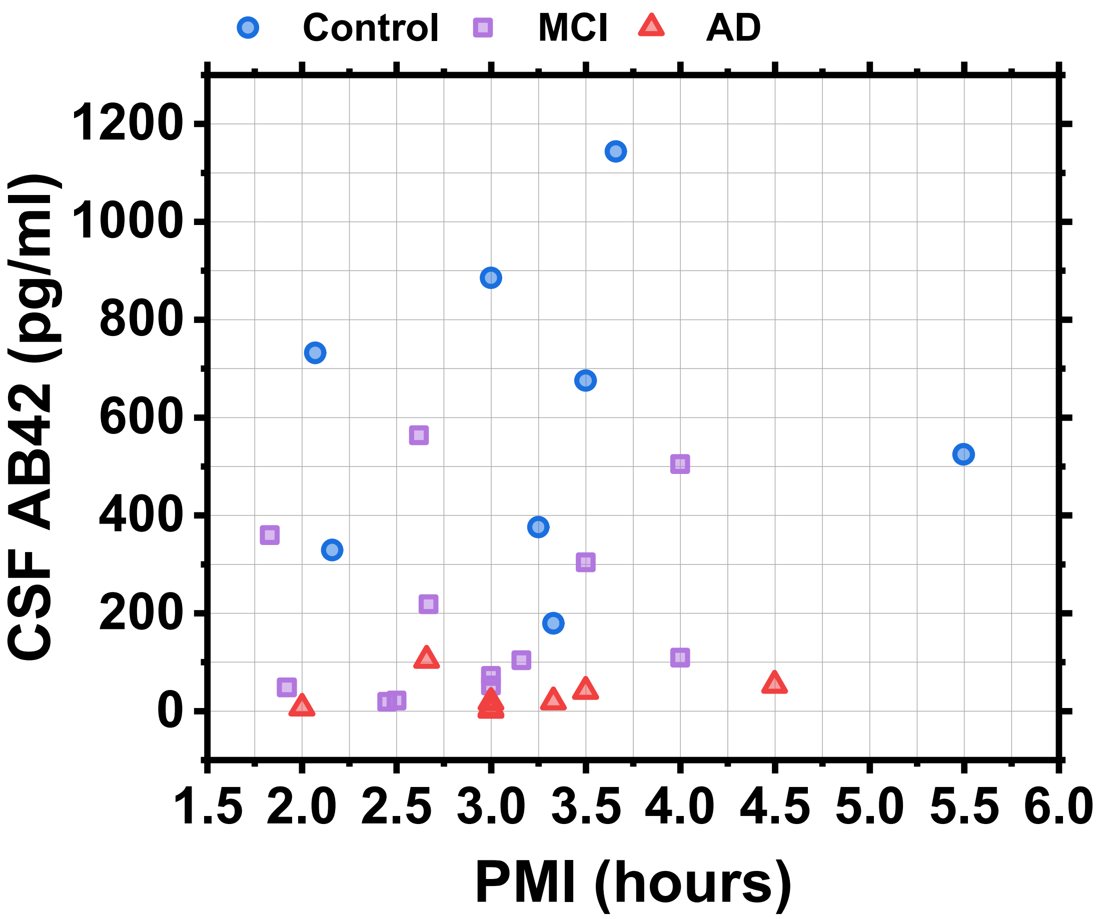

Supplementary Figure 3. FLOWER assay CSF AB42 vs. postmortem interval (PMI). Spearman correlation coefficient: ρ=0.11, P=0.55.

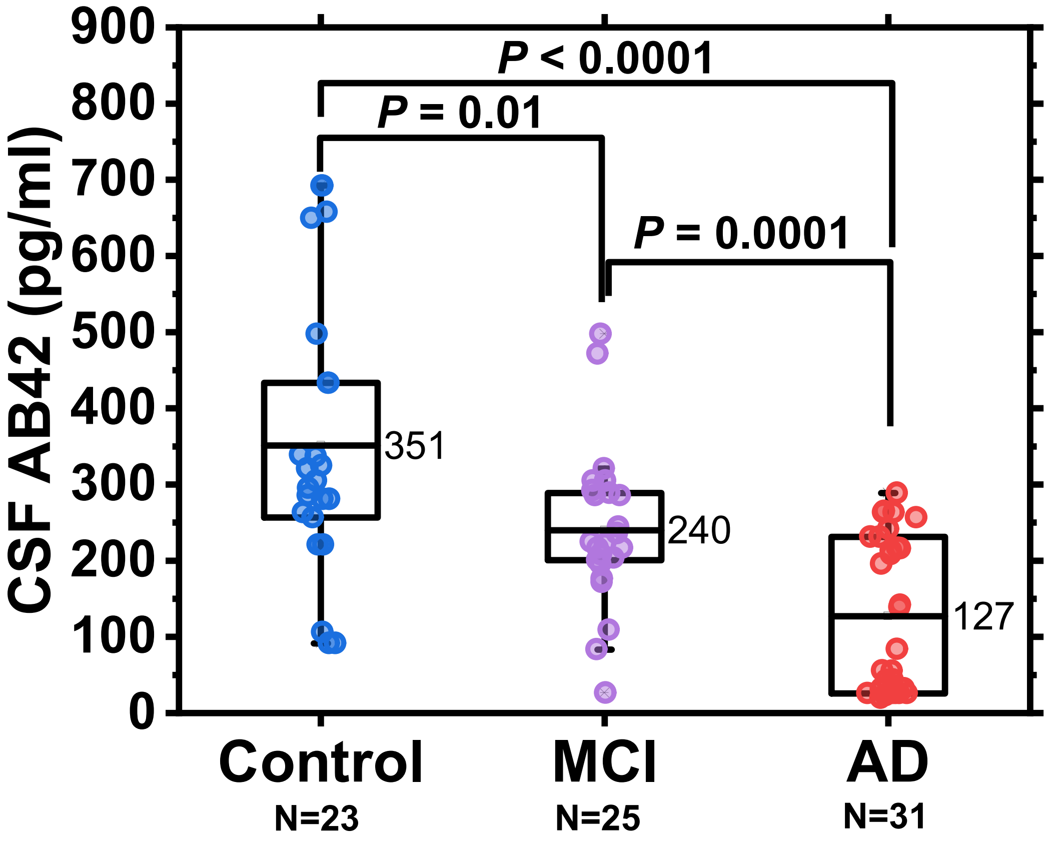

Supplementary Figure 4. CSF AB42 ELISA. CSF samples from control, MCI, and AD subjects were screened using an ultrasensitive, human AB42 ELISA kit from Invitrogen (ThermoFisher, Cat# KHB3544). Boxes show the mean, first, and third quartile. Whiskers show 1.5x interquartile range. Box labels show the mean CSF AB42 for each group. One-way ANOVA was used to determine statistical significance between the 3 groups.

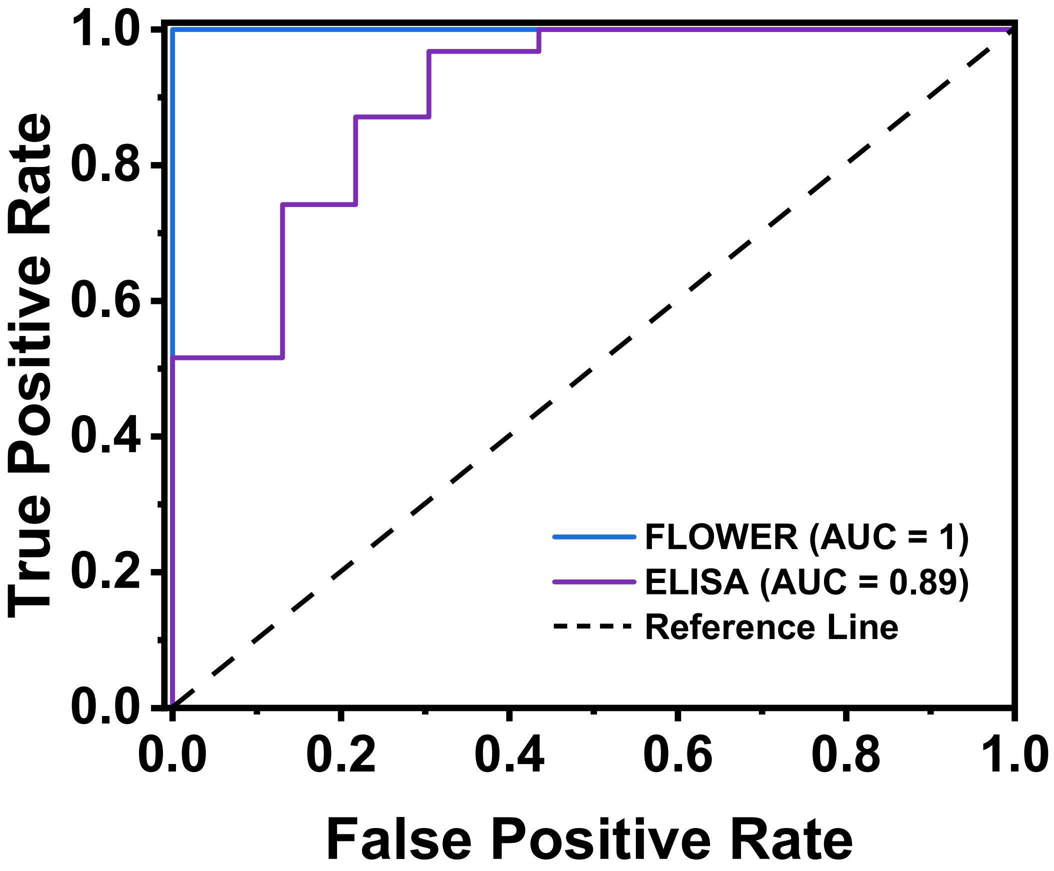

Supplementary Figure 5. ROC curve for FLOWER and ELISA using only control and AD diagnosed participants. Control participants were categorized as the negative group while AD diagnosed participants were categorized as the positive group.

Supplementary Reference

1. Zhang, T., Nagel-Steger, L. & Willbold, D. Solution-Based Determination of Dissociation Constants for the Binding of Aβ42 to Antibodies. *ChemistryOpen* **8**, 989–994 (2019).
